# Tracking and predicting COVID-19 radiological trajectory on chest X-rays using deep learning

**DOI:** 10.1038/s41598-022-09356-w

**Published:** 2022-04-04

**Authors:** Daniel Gourdeau, Olivier Potvin, Patrick Archambault, Carl Chartrand-Lefebvre, Louis Dieumegarde, Reza Forghani, Christian Gagné, Alexandre Hains, David Hornstein, Huy Le, Simon Lemieux, Marie-Hélène Lévesque, Diego Martin, Lorne Rosenbloom, An Tang, Fabrizio Vecchio, Issac Yang, Nathalie Duchesne, Simon Duchesne

**Affiliations:** 1 Université Laval, Quebec, Canada; 2 Université McGill, Quebec, Canada; 3 Université de Montréal, Quebec, Canada; 4 IRCCS San Raffaele Roma, Rome, Italy

**Keywords:** Radiography, Computer science

## Abstract

Radiological findings on chest X-ray (CXR) have shown to be essential for the proper management of COVID-19 patients as the maximum severity over the course of the disease is closely linked to the outcome. As such, evaluation of future severity from current CXR would be highly desirable. We trained a repurposed deep learning algorithm on the CheXnet open dataset (224,316 chest X-ray images of 65,240 unique patients) to extract features that mapped to radiological labels. We collected CXRs of COVID-19-positive patients from an open-source dataset (COVID-19 image data collection) and from a multi-institutional local ICU dataset. The data was grouped into pairs of sequential CXRs and were categorized into three categories: ‘Worse’, ‘Stable’, or ‘Improved’ on the basis of radiological evolution ascertained from images and reports. Classical machine-learning algorithms were trained on the deep learning extracted features to perform immediate severity evaluation and prediction of future radiological trajectory. Receiver operating characteristic analyses and Mann-Whitney tests were performed. Deep learning predictions between “Worse” and “Improved” outcome categories and for severity stratification were significantly different for three radiological signs and one diagnostic (‘Consolidation’, ‘Lung Lesion’, ‘Pleural effusion’ and ‘Pneumonia’; all *P* < 0.05). Features from the first CXR of each pair could correctly predict the outcome category between ‘Worse’ and ‘Improved’ cases with a 0.81 (0.74–0.83 95% CI) AUC in the open-access dataset and with a 0.66 (0.67–0.64 95% CI) AUC in the ICU dataset. Features extracted from the CXR could predict disease severity with a 52.3% accuracy in a 4-way classification. Severity evaluation trained on the COVID-19 image data collection had good out-of-distribution generalization when testing on the local dataset, with 81.6% of intubated ICU patients being classified as critically ill, and the predicted severity was correlated with the clinical outcome with a 0.639 AUC. CXR deep learning features show promise for classifying disease severity and trajectory. Once validated in studies incorporating clinical data and with larger sample sizes, this information may be considered to inform triage decisions.

## Introduction

The current outbreak of Severe Acute Respiratory Syndrome Coronavirus 2 (SARS-CoV-2) and the subsequent pandemic of coronavirus disease (COVID-19) are imposing a substantial stress on healthcare systems worldwide. In the majority of COVID-19 cases admitted to intensive care units (ICU)1 for respiratory distress and hypoxaemia, endotracheal intubation and ventilation are the main treatment options. The high number of infected patients has highlighted the need for more precise decision support systems for determining the need for and prognosis after ventilation, especially in healthcare networks where there is a risk of overwhelming system capacity. The recent surviving sepsis campaign recommendations do not make any specific recommendation about this triage decision making^1^. Clinical prediction rules are therefore required to help caregivers during this delicate but necessary decision making process, and these rules should be based in part on the prognosis of possible outcomes^2^. While imaging is not indicated for diagnostic purposes in COVID-19, the use of chest radiography to inform prognosis was recommended by Rubin et al. in the consensus statement of the Fleischner Society: “in a resource-constrained environment, imaging is indicated for medical triage of patients with suspected COVID-19 who present with moderate-severe clinical features and a high pre-test probability of disease”^3^. This recommendation rests on radiological findings for COVID-19, already reported in adults^4–7^. For CT imaging, they comprise (a) bilateral, subpleural, and peripheral ground-glass opacities; (b) crazy paving appearance (ground glass opacities and inter-/intra-lobular septal thickening); (c) air space consolidation; (d) bronchovascular thickening; and (e) traction bronchiectasis. COVID-19 appearance on CXR was reported more recently, with a handful of reports focusing specifically on anterior-posterior (AP CXR) at the bedside, the most common form of imaging in ICUs. CXR may be normal in early or mild disease, but commonly shows abnormal findings in patients requiring hospitalization, in 69% of patients at the time of admission, and in 80% of patients sometime during hospitalization^8^. Most frequent CXR findings are consolidation (59% of patients) and ground glass opacities (41%)^8,9^, with a peripheral and lower zone distribution, that are commonly bilateral or multilobar that tend to be patchy and asymmetric. Pneumothoraces are rare. The main finding over time on CXR was consolidation. These findings are not specific however, as they are similar to other causes of coronavirus and other viral pneumonias^10^.

It has been shown that radiological findings in CT^11^ and CXR imaging^12–14^ are correlated with disease outcome. Therefore, the ability to assess a patient’s radiological severity, track its evolution over multiple CXRs, and predict its future radiological trajectory is a medically sound basis for a COVID-19 prognosis tool. Given the critical nature of the triage decision, it is imperative that as much relevant information as possible be extracted from all available data. This information can help assess the risk of mortality, determine priority for initiating ventilation, determine improvements in condition and predict probable clinical trajectory, all of which must be considered in the intervention decision^15^.

We hypothesize that deep learning can be harnessed to analyze CXRs and extract radiological features in a reproducible and quantitative manner. Such models could be integrated in the clinical workflow to provide additional, actionable data in triage situations. This is supported by the number of successful application of AI and deep learning techniques for COVID-19 management.

### Related Works

Notably, prognosis^16^ and diagnosis^17–19^ for COVID-19 patients using CT scans have reported good results in terms of accuracy and patient stratification. However, in most of the world, CT scans are not part of the standard of care in COVID-19 patients—especially in ICUs—and CXR is the modality of choice. As such, many authors have also developed applications using CXR. For instance, differential diagnosis (Healthy, pneumonia or COVID-19) of COVID-19 positive patients can be achieved^20–22^ with accuracies reaching over 90% using deep networks such as the Xception architecture. Disease severity can be assessed in an objective way using radiology^23–25^ which is useful to quickly assess the pulmonary involvement of the disease; and the potential for adverse events can be predicted (such as death, intubation or need for oxygenation) to help direct future treatments^26^ Finally, ventilation need can be predicted in the near future for hospital-admitted patients^27^ with over 90% accuracy. These cases demonstrate that CXRs possess enough information to predict clinical developments in the near future (around three days ahead of the event). It should be noted however that in these works, adverse events prediction and radiological severity are always treated as two separate endpoints. However, they are likely to be correlated as the clinical degradation of the patient should be reflected in the radiological signs on CXR; this forms the first contribution of this article.

Most attempts at COVID-19 diagnosis or prognosis have used end-to-end deep learning methods. However, this comes at a high risk of overfitting when small COVID-19 datasets are used. Hence, alternatives for end-to-end methods are desired. Of particular interest is the combination of transfer learning and classical machine learning algorithms^18^ where machine learning algorithms were trained on the output of a convolutional network trained on COVID-19 images, or the use of radiomics as a feature extractor^19^. Another interesting avenue to limit overfitting is deep transfer learning on CXR datasets such as the CheXpert dataset^28^ to create a CXR-specific feature extractor, using supervised or contrastive learning^26^. The classifier layer is then fine-tuned jointly with the feature extractor to approach a prediction task with a dataset of>5000 images. Considering that fine-tuning deep networks is still at risk of overfitting on smaller datasets, an approach using deep transfer learning to extract general CXR pathological features that are not specific to COVID-19 would be valuable. Classical machine learning can then approach the task using these features without re-training on COVID-19 patients. This is the second major contribution of our article.

### Study contributions

A common trait among the litterature is that the radiological severity evaluation and adverse events prediction are typically viewed as two separate tasks. In this work, adverse events prediction is approached as a worsening of the radiological severity observed on successive CXRs. This paradigm shifts the target from discrete clinical decisions to a continuous spectrum of disease progression. The change in the extent of the disease in future imaging is then defined as the radiological trajectory. Knowledge of the future radiological trajectory of a patient could be useful information for a physician to make the best decision for a patient given its correlation to the clinical outcome. Towards this goal, we first demonstrate that a pre-trained non-COVID feature extractor is suitable to extract useful information with no additional training on COVID-19 CXRs. Then, we use this feature extractor on COVID-19 CXR datasets to train models that predict the disease’s severity and future radiological trajectory. The main contributions of this article are therefore as follows:

- We show that a deep learning model trained to extract convolutional features from non-COVID chest X-rays (CXR) can extract useful information without re-training on COVID-19 CXRs;
- We propose the prediction of the radiological trajectory as a continuous alternative to the binary endpoint of adverse events prediction;
- The feature extractor was used on both a convenience, open access dataset and a systematic, multi-institutional dataset. We demonstrate that these features had the ability to track severity and predict the radiological trajectory over a large spectrum of disease presentations.

## Materials and method

### Study design

This is a retrospective study of a large dataset of CXRs (open access), a convenience series of COVID-19 cases (open access), and a multi-institutional dataset of clinical ICU cases. The whole study was approved by the ethics committee of all participating hospitals (CIUSSS-Capitale-Nationale certificate #MP-13-2020-2025) and was performed according to the relevant guidelines and regulation. Informed consent was waived due to the retrospective nature of the study. This study is conducted and reported based on the STARD criteria^29^.

### Datasets

#### ChexPert dataset

*Training the feature extractor* We used as training set the open “CheXpert” chest X-ray dataset from Stanford Hospital, comprised of 224,316 X-ray images taken from 65,240 unique patients (aged 60.4 ± 17.8 years (mean ± standard deviation); 132,636 CXRs from men/90,777 CXRs from women)(Table 1)^28^. The CheXpert database was originally extracted from the Stanford Hospital PACS system with the assistance of text mining from the associated radiological reports using natural language processing. The dataset includes both posterior-anterior, anterior-posterior and lateral images. None were from COVID-19 positive patients.

*Feature extractor validation* The validation set (n = 234) for deep learning feature extraction was selected at random within the 500 validation set studies that forms part of the CheXpert dataset (https://stanfordmlgroup.github.io/competitions/chexpert/)^28^. The latter was composed of randomly sampled studies from the full dataset with no patient overlap. Three board-certified radiologists from the CheXpert team individually assigned radiological findings and diagnoses to each of the studies in this validation set.

#### COVID-19 image data collection

The open-access COVID-19 dataset was curated from a convenience sample of 77 patients with sequential AP CXRs (aged 58.1 ± 15.8 years; 79 men, 34 women, 11 not reported) (Table 1). There were 124 CXR pairs with less than a 7 days span between them, accessible in the MILA COVID-19 image data collection (https://github.com/ieee8023/covid-chestxray-dataset/). A full list of cases, including links to original sources, is included in Supplementary Material 1. Data was downloaded on August 18, 2020.

*Dataset splitting* 24 patients from the COVID-19 image data collection were randomly allocated to the testing set for a total of 37 CXR pairs. The remaining 53 patients from the COVID-19 image data collection were included in the training set for a total of 87 CXR pairs.

#### COVID-19 ICU dataset

We collected imaging and clinical data (outcome, risk factors, demographics) for all adult patients that were admitted at the ICU for confirmed COVID-19 and were treated using intubation and mechanical ventilation in five Quebec hospitals (Centre hospitalier universitaire de Quebec; Hotel-Dieu de Levis; Institut universitaire de cardiologie et pneumologie de Quebec; Jewish General Hospital, Centre hospitalier universitaire de Montreal) between March 2020 and February 2021. The canonical index test for COVID-19 presence was the polymerase chain reaction test. CXRs were taken irregularly (depending on the institution) prior to and after intubation as part of the standard of care. The post-intubation CXR was the only reliably collected timepoint. Only 123 patients (aged 65.7 ± 10.0 years; 346 men, 152 women, 0 not reported) had multiple CXRs to assess radiological progression, for a total of 498 CXR pairs (Table 1).

*Dataset splitting* The patients in the dataset were randomly split between testing and training using stratified sampling on the basis of outcome (Death/Hospital discharge). The number of patients is not equal between training and testing because the groups were assigned prior to this study and some patients had only a single image and could not be used.

**Table 1:** Group demographics information.

Group	N	Age	Sex (M/F/unknown)
CheXpert	223,414	60.4 ± 17.8	136,636/90,777/0
COVID-19 image data collection	124	58.1 ± 15.8	79/34/11
Worse	76	60.0 ± 16.3	47/22/7
Stable	24	49.9 ± 12.9	17/3/4
Improve	24	59.8 ± 14.1	15/9/0
ICU dataset	498	65.7 ± 10.0	346/152/0
Worse	110	65.5 ± 10.9	78/32/0
Stable	327	65.6 ± 9.9	224/103/0
Improve	61	66.7 ± 9.2	44/17/0

### Index test and reference standard

By patient, sequential AP CXRs with less than a week of time difference were grouped into pairs. The primary outcome for each pair of sequential AP CXRs was a categorical classification of radiological evolution (“Worse”; “Stable”; and “Improved”) and defined based on the radiological case history provided with the open dataset as well as the images themselves (Supplementary Material 1). Evaluation of the severity of the disease was also performed by the radiologists for each image. The adopted severity scale was “No disease”; “Mild”; “Severe”; and “Critical”. The history was performed by certified radiologists at the centers providing cases. The categorical and severity classifications was done by two authors on the COVID-19 image data collection ([N.D.] 25 years practice; [N.D.] fourth year residency) for indications regarding radiological outcome. Only the categorical classification was performed for the ICU dataset by a third radiologist ([N.D.] third year residency). The readers were blind to the outcome. If, when compared to the first CXR of a pair, additional findings (e.g. new lung opacities, or increase in lung opacities already present) were noted in the second CXR, then the pair was categorized as “Worse”. If no change was reported, it was labeled “Stable”; and if improvements were described, the category was “Improved”.

#### COVID-19 image data collection

There was a total of 124 such pairs in the COVID-19 imaging data collection, given that some patients received more than two CXRs. There were 76 pairs of successive CXR studies in the “Worse” outcome category, 24 in the “Stable”, and 24 in the “Improved” outcome categories (Table 1). Mean age for the “Worse” outcome group was 60.0 ± 16.3 years (mean ± standard deviation) (47 men, 22 women, 7 not reported); for the “Stable” group 49.9 ± 12.9 years (17 men, 3 women, 4 not reported); and the “Improved” group 59.8 ± 14.1 (15 men, 9 women, one not reported).

#### COVID-19 ICU dataset

**Figure 1:**
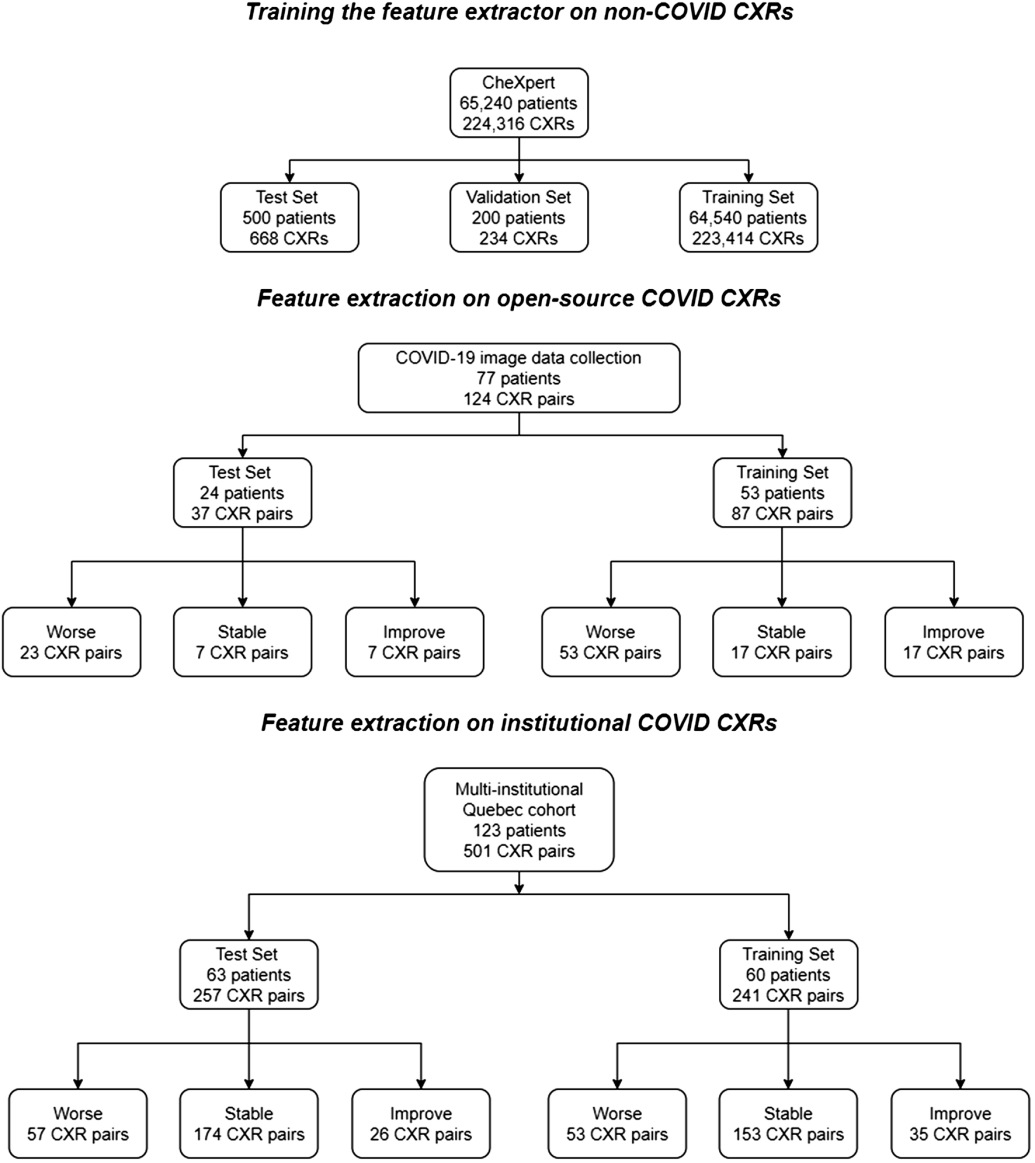
Study flowchart. Some patients may have CXR pairs in more than one outcome category.

There was a total of 498 such pairs in the COVID-19 imaging data collection. There were 110 pairs of successive CXR studies in the ‘Worse’ outcome category, 327 in the ‘Stable’, and 61 in the ‘Improved’ outcome categories (Table 1). Mean age for the ‘Worse’ outcome group was 65.5 ± 10.9 years (mean ± standard deviation) (78 men, 32 women); for the ‘Stable’ group 65.6 ± 9.9 years (224 men, 103 women); and the ‘Improved’ group 66.7 ± 9.2 (44 men, 17 women). Figure 1 summarizes the distribution of patients.

### Input variables and deep learning algorithm

We trained a deep learning model for feature extraction, taking as input all single-view chest radiographs of the training CheXpert dataset (regardless of patient position) and providing as output the probability of nine radiological findings and one radiological diagnostic category (“Pneumonia”, used as a label by the CheXpert authors to represent images that suggested primary infection as the diagnosis). The findings were defined by certified radiologists in CheXpert. We removed the following radiological findings from the training set, given their irrelevance to the purpose of our study: “No Findings”; “Fracture”; “Support Devices”. We further removed “Pneumothorax”, given its low occurrence in COVID-19. We used a DenseNet121 architecture for all our experiments as it was determined by Irving et al.^28^ to achieve the best results on the CheXpert dataset. CheXpert images were fed into the network with pretrained weights on Imagenet with a size of 320 $\times$ 320 pixels without any frozen layers. Data augmentation was performed by randomly cropping the input images to 290 $\times$ 290. Images were histogram-equalized using OpenCV. We used the Adam optimizer with default $\beta$-parameters of $\beta _1$ = 0.9, $\beta _2$ = 0.999 and learning rate of $1 \times 10^{-4}$ which was fixed for the duration of the training. Batches were sampled using a fixed batch size of 16 images. We used a weighted binary cross-entropy loss function to account for class imbalance. In this scheme, the cross entropy results are divided by the label’s prevalence in the training set to give more weight to examples in the minority classes. We also followed the U-zeroes policy from^28^, replacing the uncertain findings with negative findings. We trained for four epochs, saving checkpoints every epoch and using the checkpoint with the lowest validation loss. This trained network is subsequently used as a feature extractor by removing the fully connected layer and using the last convolutional layer as the feature vector.

### Radiological trajectory prediction

**Figure 2:**
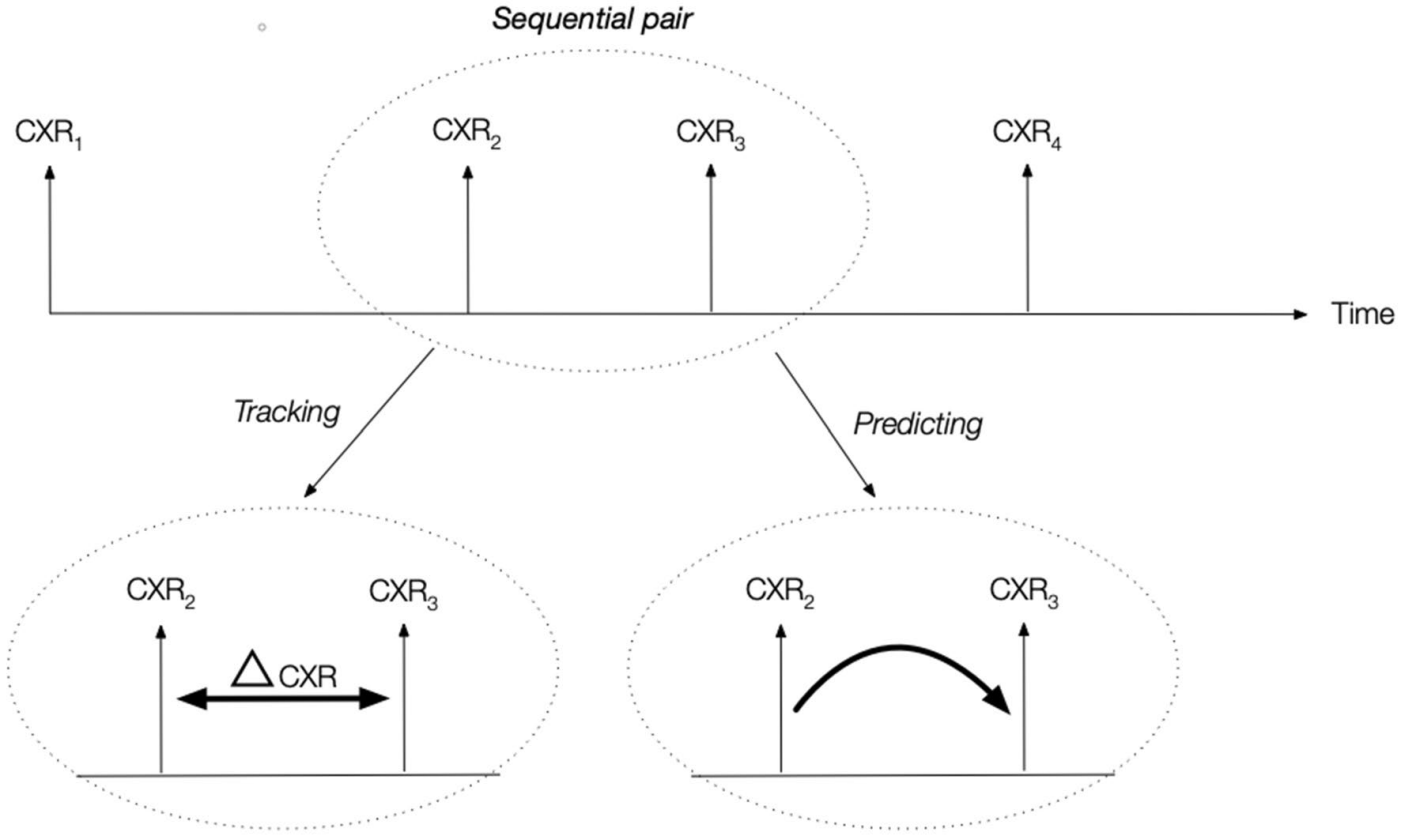
Experimental design. For each patient, the acquired CXRs formed a series of sequential pairs. For each pair (example shown for CXR2 and CXR3), an outcome was defined by judging if the radiological evolution of second CXR of the pair was worse, stable or improved compared to the first. We then tested whether the difference ($\Delta$ CXR) in radiological findings probabilities would be statistically different between outcome categories; and secondly if deep learning features from the first CXR of the pair would predict radiological evolution.

We then proceeded in testing our hypothesis as follows (Fig. 2). First was whether deep learning could track radiological evolution. We used the deep learning network as trained above to extract the findings probabilities from each CXR. We then computed the difference in findings probabilities between sequential CXRs in each pair and tested whether this difference was significant between outcome groups. We also investigated if the findings probabilities were correlated with the severity of the disease, and tested whether the probabilities were significant between severity groups.

**Figure 3:**
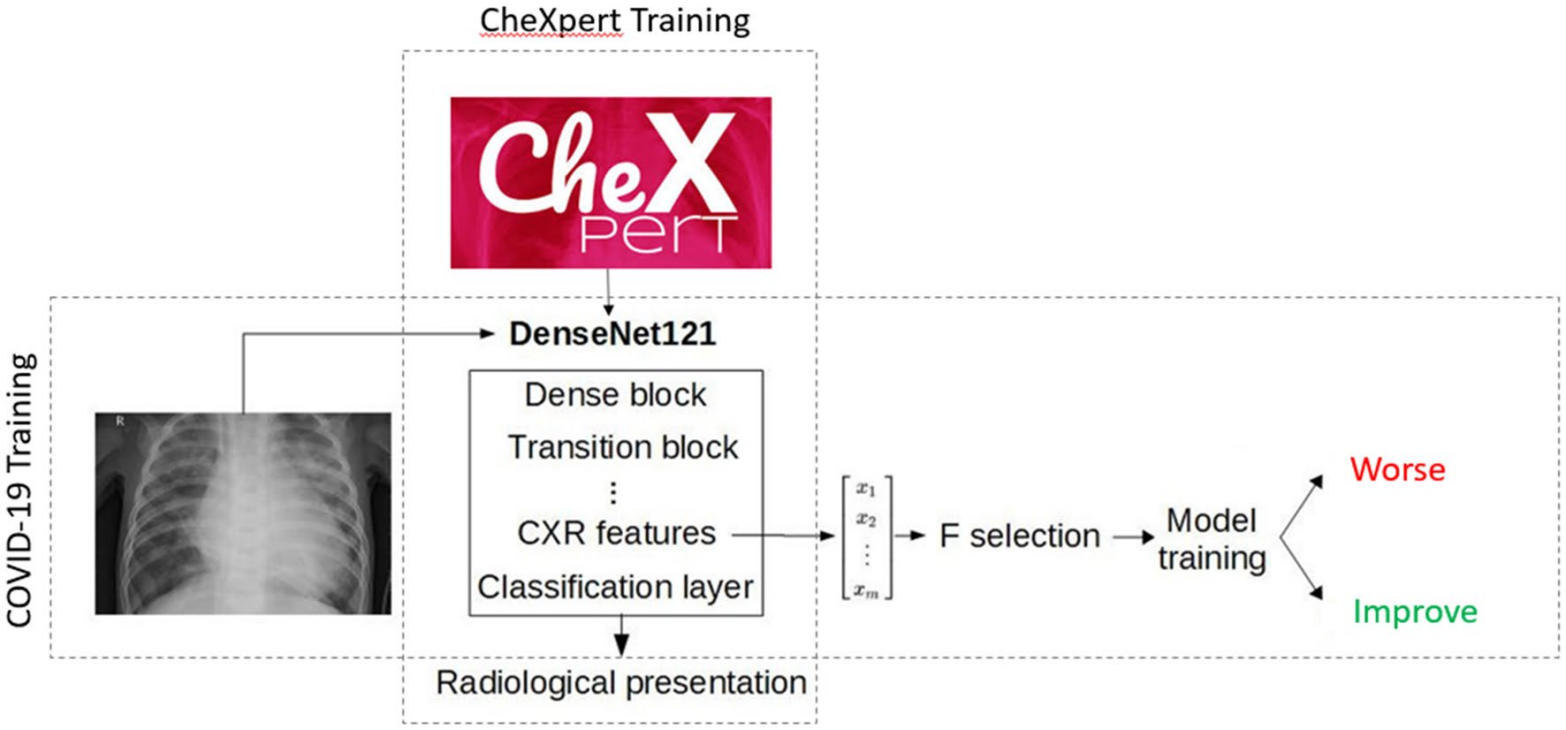
High-level description of the method. In a first step, the DenseNet121 is trained on the CheXpert dataset. Then, the network is frozen and COVID-19 CXRs are fed to the network. The features at the last convolutional layer are extracted and used as the feature vector for the COVID-19 model selection and training.

Secondly, we attempted to assess the predictive power of the extracted deep learning features, i.e. whether or not the features of the first CXR could predict the outcome category of the CXR pair (Worse or Improved) (Fig. 2). Instead of using the predicted findings or the findings probabilities from the deep learning network, we used the output of the last convolutional layer. A high-level summary of the method is shown in Fig. 3. This layer was average-pooled to a 1 $\times$ 1 size to reduce dimensionality. Data augmentation was performed at the feature extraction stage in the same way as when training the network on the CheXpert dataset. We extract features for 10 random crops per CXR in both COVID datasets, providing us with an estimate of the features robustness. Only stable features are selected by removing features with a inter-crop variance higher than a given threshold. We further reduced the dimensionality of this feature space by selecting the best N features using the ANOVA F value. The number of features (N), the variance filtering threshold, the type of classifier and the regularization coefficients were tuned using cross-validated random search. The candidate models were random forests, logistic regression and support vector classifier. We performed cross-validation using a leave-one-patient-out scheme, removing/testing all pairs associated with this patient in the learning/validation phase. We ranked and selected the candidate models using the ROC-AUC before testing. 95% confidence intervals on the model’s AUC are found by percentile bootstrapping.

In the case of predicting the severity of the disease on the current CXR, the same model selection methodology was also used. In that situation, the candidate models were random forest regression, polynomial regression or linear regression and the best candidate was selected on the basis of minimizing the average error.

This model selection methodology is identically applied to both COVID-19 datasets without mixing. The severity regression model is trained only on the COVID-19 image data collection as the severity evaluation was not performed on the ICU dataset.

### Statistical analysis

Demographics were expressed as mean (standard deviation) in years, and differences between outcome groups were tested using the SciPy library. Statistical analysis of the deep learning algorithm consisted in calculating the area under the (receiver operating) curve (AUC) for the determination of label learning on the training set; and Mann-Whitney tests to compare results between outcome groups. We used a bilateral *P* < 0.05 threshold for significance and calculated effect size (Cohen’s d) for each output.

### Software

Deep learning feature extraction was done in Python using the PyTorch library (version 1.4). Our source code will be available after acceptance in the following GitHub repository: https://github.com/medicslab/COVID-19-public.

## Results

### Training the DenseNet on CheXpert dataset

**Figure 4:**
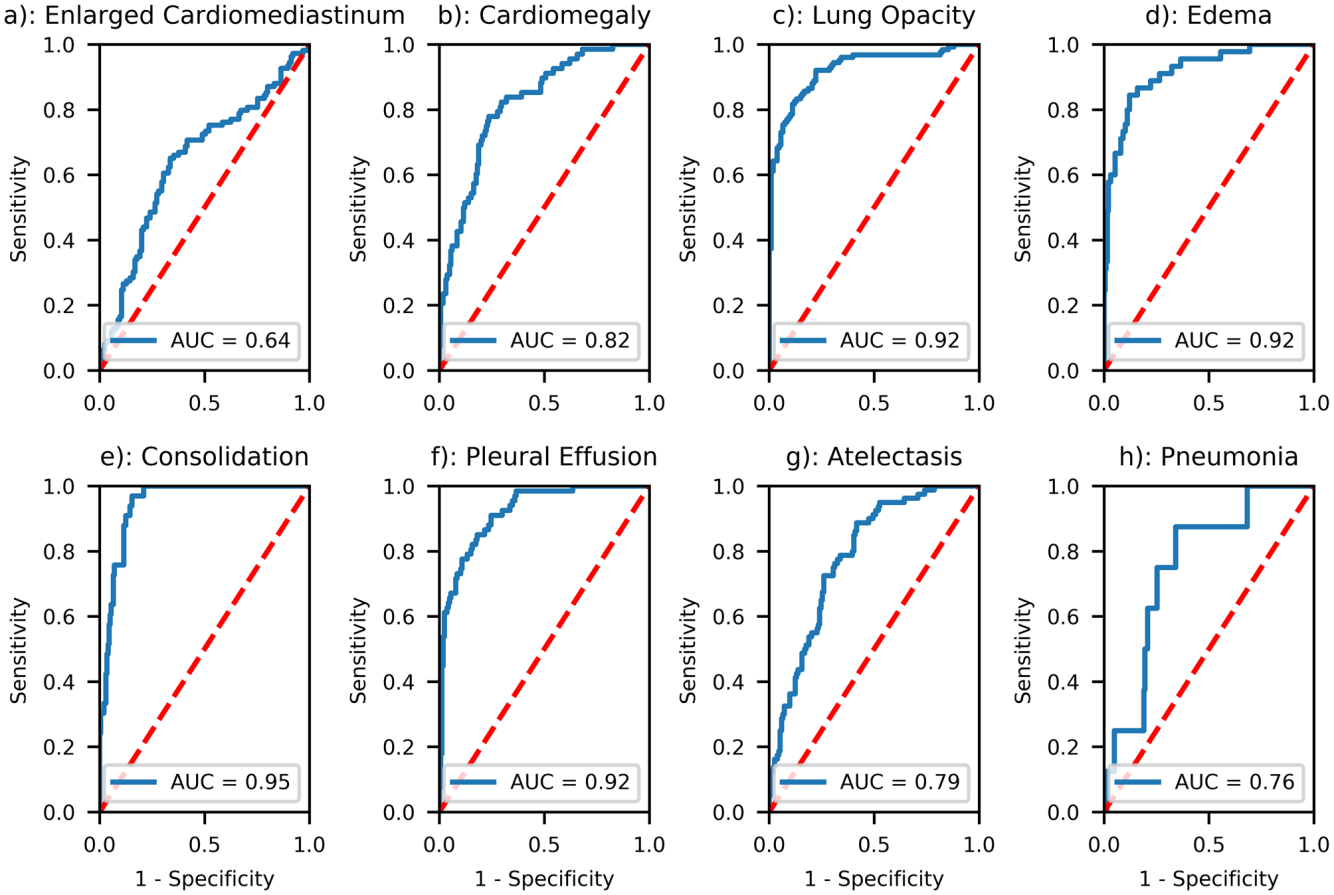
Results of the deep learning architecture trained on CheXpert for seven radiological findings (**a**–**g**) and one radiological diagnosis (**h**) on a separate 234-cases test dataset, selected at random within the 500 test set studies of the CheXpert dataset (cf. Irvin et al. for details). The latter was composed of randomly sampled studies from the full dataset with no patient overlap. Three board-certified radiologists individually annotated each of the studies in this test set.

**Figure 5:**
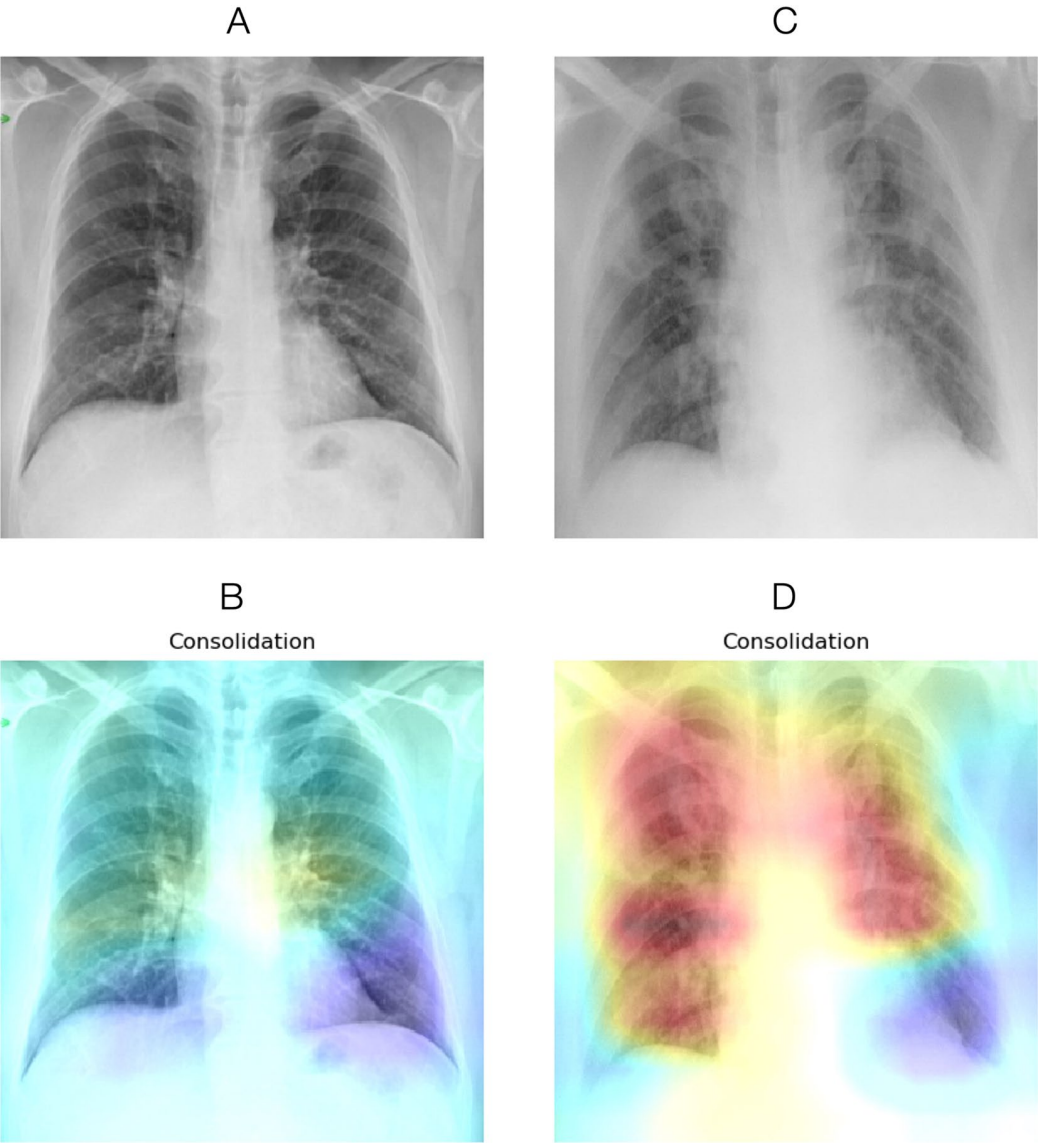
Original CXRs and class activation maps for a random patient in the COVID-19 dataset. (**A**) CXR at admission, with (**B**) overlaid activation map for the most activated radiological finding (“Consolidation”). (**C**) CXR four days later, with a worsening radiological presentation. The regions activated for the same finding (**D**) now encompass a larger area as the disease has progressed.

We successfully trained the deep learning algorithm with the aforementioned architecture to extract salient radiological findings, attaining results comparable to those from the original authors of the CheXpert series (Fig. 4) with AUCs ranging from 0.64 (“Enlarged Cardiomediastinum”) to 0.95 (“Consolidation”). We were unable to ascertain AUCs for two radiological findings (“Lung Lesion”, “Pleural—Other”) due to a lack of sufficient number of cases in the validation dataset. We generated a class-activation map^30^ for the highest-activated radiological sign (“Pneumonia:) on a random COVID-19 patient for illustrative purposes (Fig. 5).

### Outcome prediction

**Figure 6:**
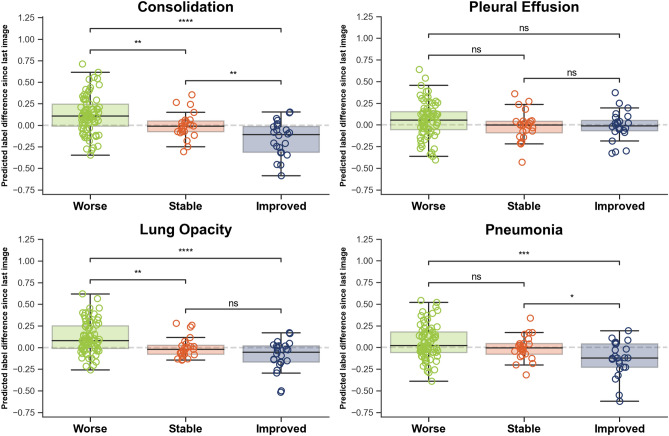
Boxplots show significant inter-group differences between Worse vs Improved (*P* < 0.05, indicated by symbol *) in predicted imaging findings: “Consolidation”, “Lung opacity”, and diagnosis of pneumonia.

**Figure 7:**
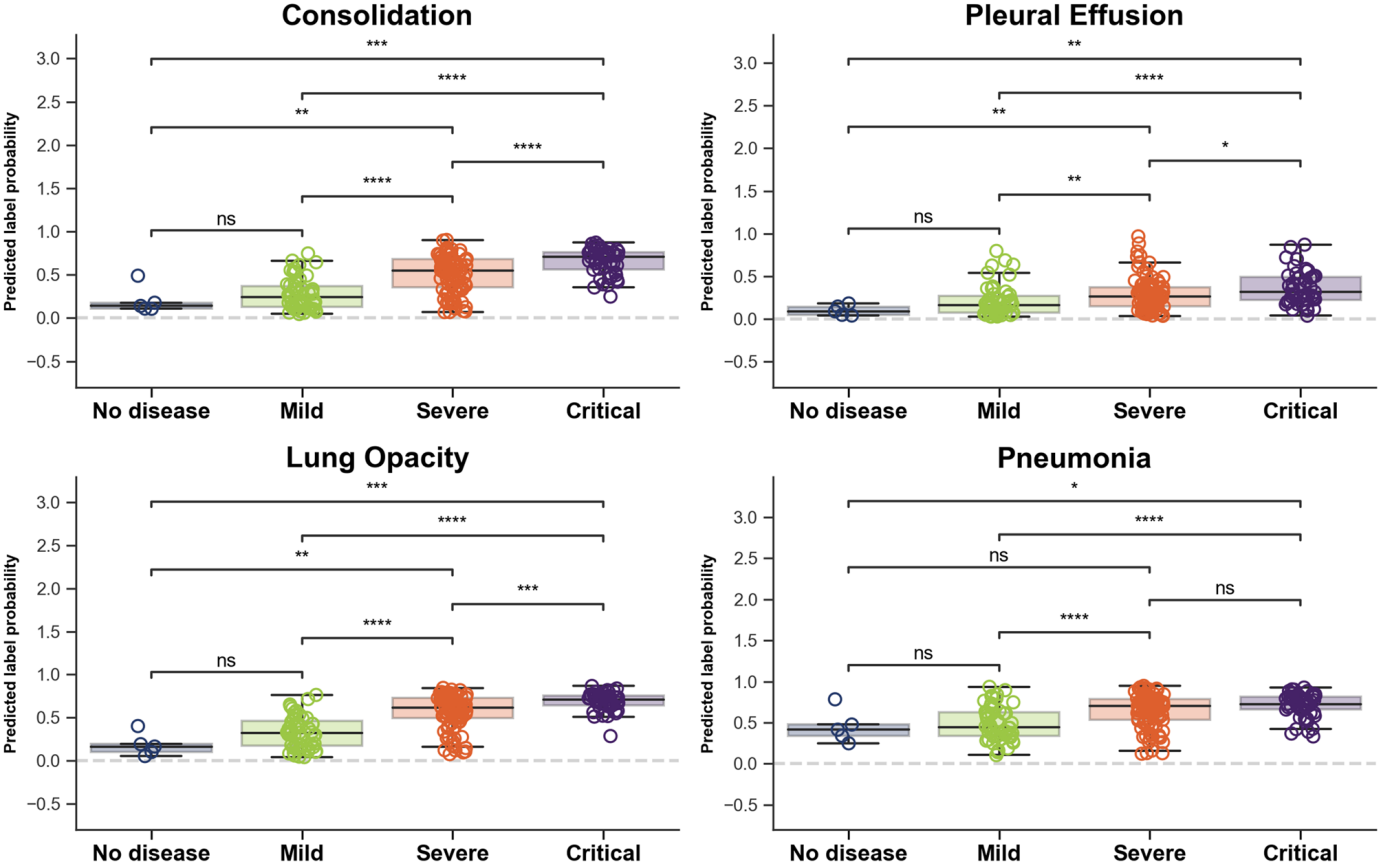
Boxplots show significant inter-group differences between Severity groups (*P* < 0.05, indicated by symbol *) in predicted imaging findings: “Consolidation”, “Lung opacity”, Pleural effusion and diagnosis of pneumonia.

Testing whether deep learning features could track disease trajectory and severity, we applied the learned classifier to the whole COVID-19 dataset, extracted radiological signs probabilities, and computed the differences between sequential CXRs. The four main findings related to COVID-19 are shown in Fig. 6 for each outcome group and in Fig. 7 for each severity group. There were significant inter-group differences (Mann-Whitney *P* < 0.05) for three radiological findings and diagnoses (“Consolidation”, “Lung Opacity” and “Pneumonia”) in terms of both evolution and severity and a significant difference for the “Pleural effusion” sign when considering severity. The Cohen’s d effect sizes for evolution and severity are presented in Table 2.

**Figure 8:**
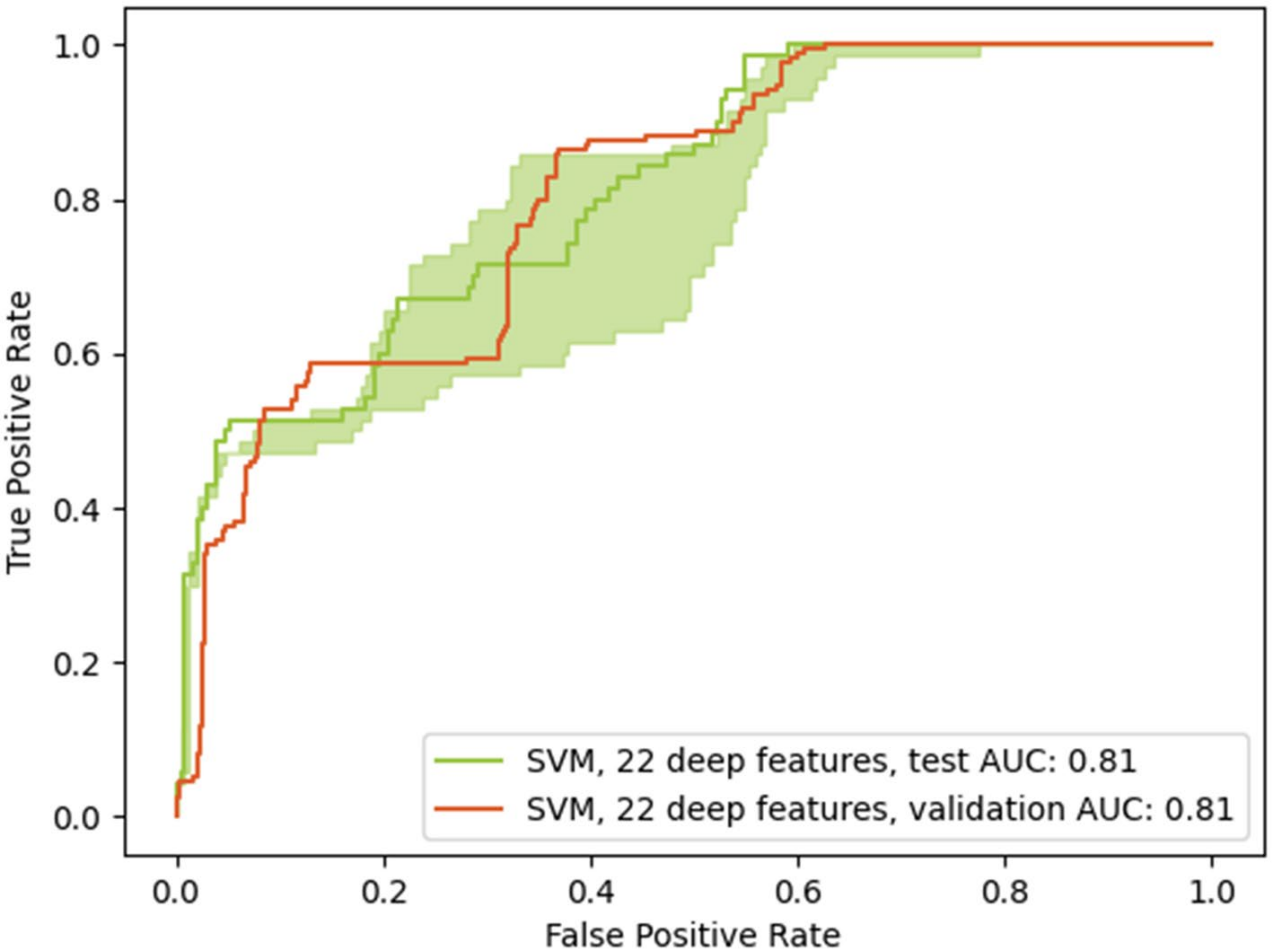
Receiver operating characteristics curves for the best performing model (validation set and applied to the testing set) for the purpose of predicting worsening of the radiological state. The chosen model was a support vector classifier with a sigmoid kernel and using 22 convolutional features. The testing AUC is up to 0.81, equivalent to the validation AUC of 0.81. The green shaded area corresponds to the 95% confidence interval for the testing AUC and ranges from 0.74 to 0.83 AUC.

**Figure 9:**
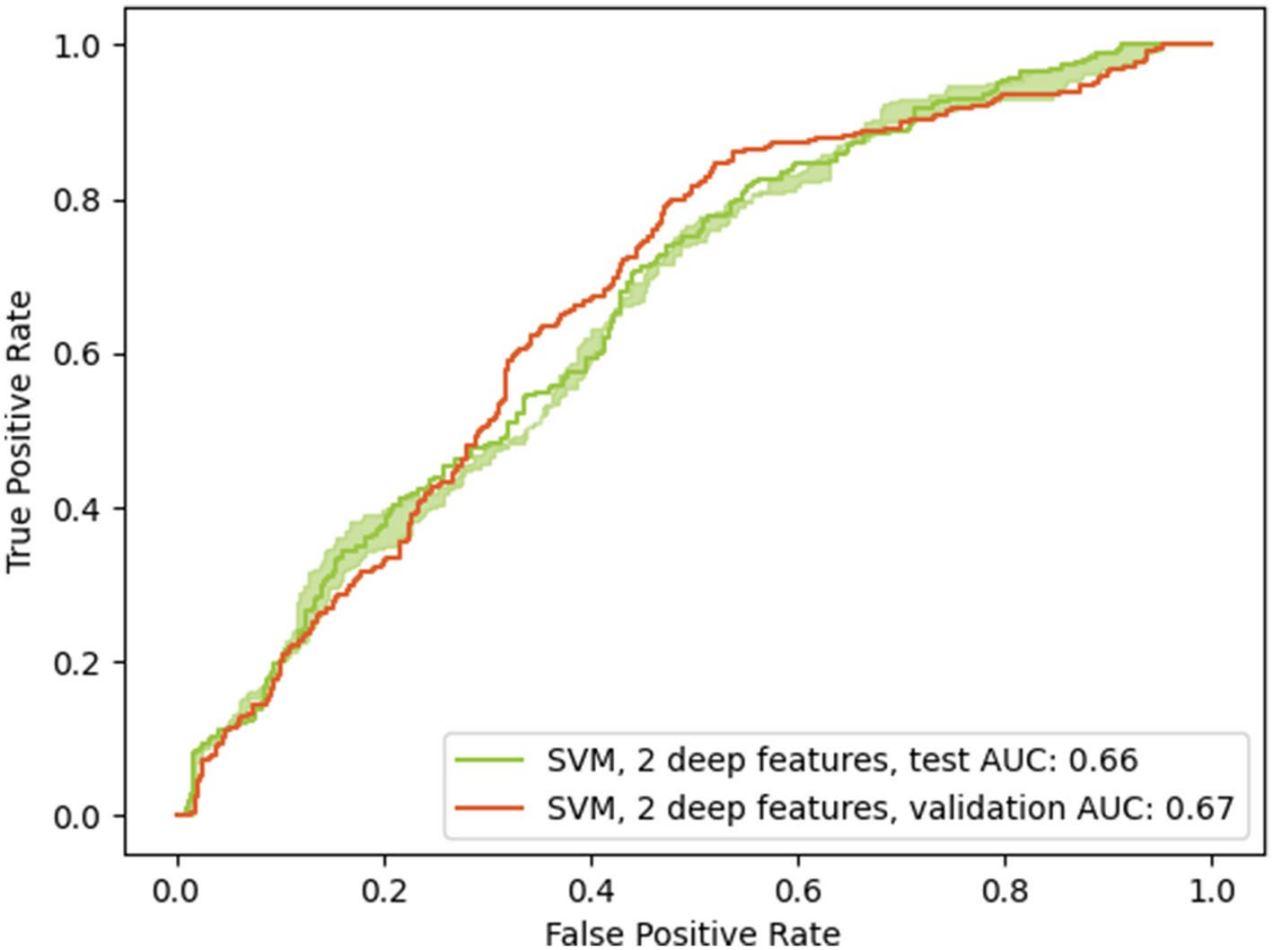
Receiver operating characteristics curves for the best performing model (validation set and applied to the testing set) for the purpose of predicting worsening of the radiological state. The chosen model was a support vector classifier with a sigmoid kernel and using 2 convolutional features. The testing AUC is up to 0.66, equivalent to the validation AUC of 0.67. The green shaded area corresponds to the 95% confidence interval for the testing AUC and ranges from 0.64 to 0.67 AUC.

The potential of deep learning to predict the radiological trajectory was confirmed in both datasets. Tuned hyperparameters are shown in Table 3. For the COVID-19 image data collection, the last convolutional layer was reduced from 1,024 to 22 features and the selected model was a Support Vector Classifier with a sigmoid kernel. Similarly, the chosen classifier on the ICU dataset was also a Support Vector classifier with a sigmoid kernel, but using only 2 features instead of 22. Good generalization was obtained from the validation set to the testing set in both cases, with equivalent performances (0.81 compared to 0.81 and 0.66 compared to 0.67). Figs. 8 and 9 present the ROC curves for both models. The green shaded area corresponds to the 95% confidence intervals, computed by percentile bootstrapping.

**Figure 10:**
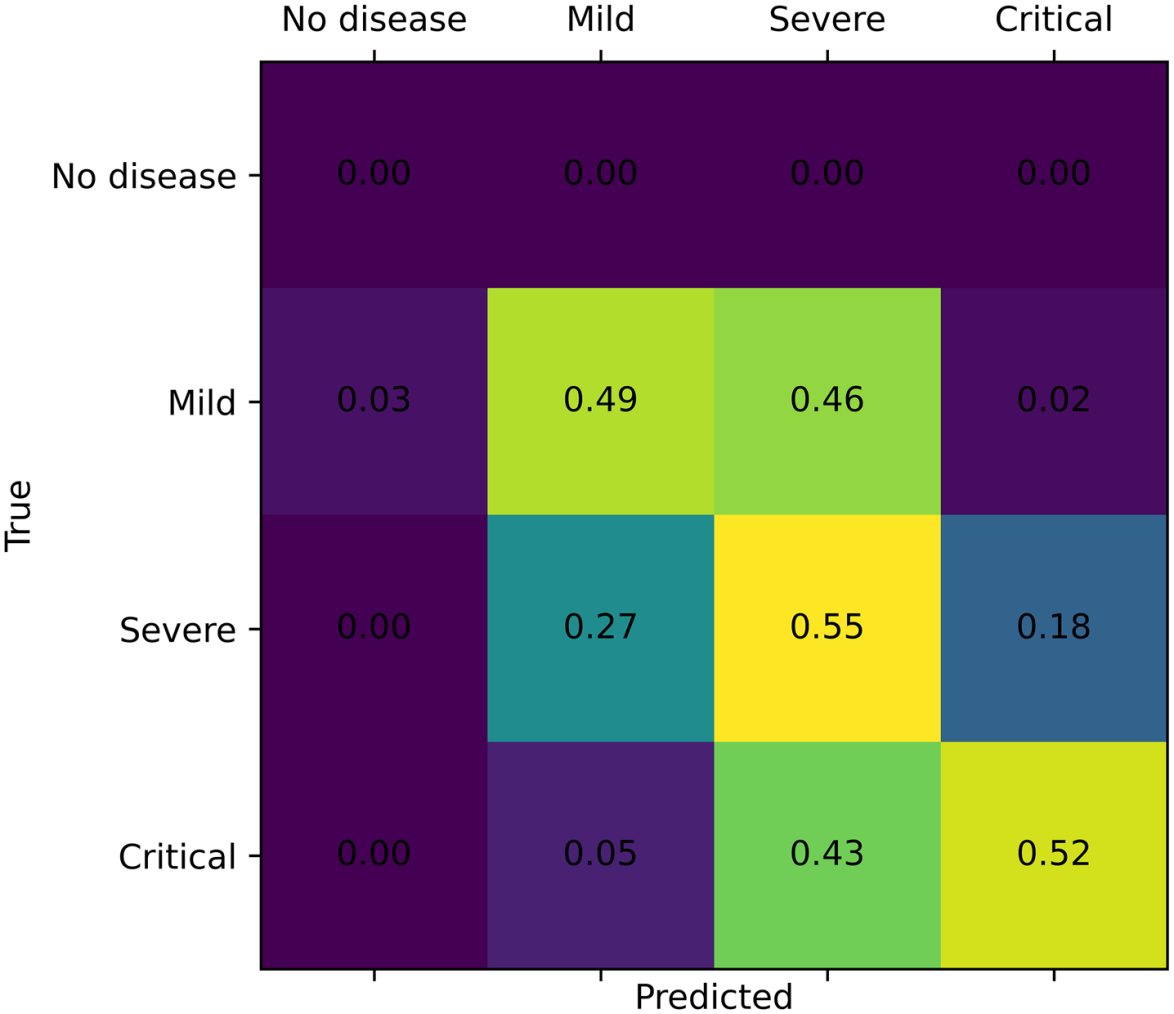
Confusion matrix (normalized) of the assessment of the severity of the current CXR using the convolutional features on the testing set. A linear regression model trained using 5 features performed best and reached a 52.3% accuracy and a 0.552 average error on the testing set.

Performances when predicting the severity of the disease on the current CXR using the convolutional features yielded best performances on the validation set when using a linear regression model applied to the best 5 features. The accuracy on the testing set when rounding to the nearest integer was 52.3% in the four-way classification of severity, with a 0.55 average error on the raw regression (an error of one would mean a misclassification of one severity step, ex. from ‘Mild’ to ‘Severe’). The confusion matrix in Fig. 10 shows good separation between the severity groups. The ‘No Disease’ severity level was not represented in the testing set.

Severity prediction trained on the COVID-19 image data collection was directly applied to the ICU dataset, with 81.6% of images labeled as ‘Critical’ and 18.4% labeled as ‘Severe’, which is consistent with an ICU-bound, intubated population. Also, the predicted severity was correlated with the clinical outcome (Death or Hospital discharge) as the predicted severity alone had a 0.639 AUC when applied to this task.

**Table 2:** Extracted deep learning feature differences between groups (Mann-Whitney).

Rx sign/Diagnosis	Worse vs Improve *P* value (Cohen’s d)	Mild vs Severe *P* value (Cohen’s d)
Consolidation	2e−6 (1.340)	6e−94 (1.16)
Lung Opacity	2e−6 (1.178)	3e−11 (1.28)
Pleural effusion	0.0806 (0.376)	0.003 (0.397)
Pneumonia	0.0004 (0.945)	2e−5 (0.764)

**Table 3:** Radiological progression classifier hyperparameters and testing performances for both datasets.

Dataset	Classifier	Selected features	C	Gamma	Test AUC	95% CI
COVID-19 image data collection	Sigmoid SVM	22	0.09	1	0.81	0.74–0.83
ICU Dataset	Sigmoid SVM	2	0.06	1	0.66	0.64–0.67

## Discussion

### Summary

Triage decisions to decide if and when patients should be admitted to the ICU and be mechanically ventilated during the current COVID-19 pandemic must be based on all available prognostic evidence. We hypothesized that deep learning analysis of baseline CXR and longitudinal changes in feature probabilities could provide objective information such as current and future radiological severity to help in these triage decisions. To this end we needed first to prove the ability of deep learning to assess imaging features and predict imaging outcomes related to the disease. Consequently, we used a deep learning architecture, pre-trained on a large CXR dataset, and able to learn image features related to nine radiological signs and one pneumonia diagnosis. We applied this algorithm to a series of sequential images from patients with suspected or proven COVID-19. The algorithm was able to significantly detect changes in the images related to either a worsening or improving outcome for the patient and predict the category from the first CXR with reasonable AUC on two different datasets. Additionally, radiological severity could be estimated from the CXRs and the model demonstrated good cross-dataset generalizability with the severity being correlated with the outcome as previously demonstrated in the litterature^12,13^.

### Findings and implications

We found that the proposed deep learning architecture was able to derive meaningful feature classes from a large yet disparate number of images. In effect the CheXpert dataset was not curated specifically for pneumonia; images were acquired in a variety of positions (e.g. anterio-posterior, posterior-anterior, and lateral views); and there were a number of non-pathologically related artefacts (e.g. various devices creating image shadows). Yet, it proved robust at extracting those deep learning features that best correlated to the radiological findings in the validation and test sets, the latter only composed of AP CXRs. The class activation maps of Fig. 5 are indicative of the process and show that the deep learning architecture is correctly focusing on relevant areas. The value of the deep learning features to inform triage decision making however lies not so much in the identification of radiological findings; this task is being done by radiologists themselves in the course of their duty. Rather, it centers on the ability to extract image features, distributed over the image, that may prove salient at the task of predicting outcome. These may be subtle, counter-intuitive, and therefore not part of the usual radiological diagnostic checklist or report; be subject to inter-reader variability; or couched in language that would vary between readers and centers. By quantitatively calculating these features, the model provides objective, repeatable estimates that may have better predictive ability than the binarized appraisal of disease status as exemplified in clinical scores such as the SMART-COP (“multilobar: yes/no”)^31^.

### Study limitations

This study has some limitations. First, the small size of the COVID-19 image data collection dataset, which inevitably must be augmented to avoid potential bias, most notably case selection, and to confirm generalizability. Nevertheless, the confidence intervals on the AUC demonstrated that the model was robust to changes in the training set. The issue of dataset bias is very problematic in most COVID-19 prognosis studies as pointed by^32,33^, which propose as a solution to identify a very specific use-case and then proceed to collect a representative dataset. To address the issue with our first dataset, we reassessed our methodology on a second, systematically collected dataset with a more homogeneous patient population. On the ICU dataset, the trained model showed lower performance (0.66 compared to 0.81) but higher generalizability and very tight confidence intervals (0.63–0.67), probably due to the simpler model that used only 2 features. An hypothesis for the lower performances is that the ICU dataset covers a narrower range of severity, where the radiological improvement and worsening is more subtle. Indeed, more patients were graded as ‘Stable’ in the ICU dataset (65%) than on the COVID-19 image data collection (19%). Interestingly, the two features used in the ICU dataset model did not overlap with the 22 used by the other model, suggesting that the features indicative of future radiological changes are dependant on the disease’s extent or severity. Next, the time duration between sequential CXRs was not uniform, which may have diminished the appraisal of the features’ sensitivity to change and the predictive ability of our model. A significant issue with the objective of predicting the future radiological course of the disease is the effect of intervention on the disease’s course. In the case of the COVID-19 image data collection, information about the treatment is scarce and very well could affect the quality of the predictions since those are dependant upon the care that the patient receives. In this work, since the images came from hospital-admitted patients, the predictions of the model are conditional upon the patient receiving standard of care for their current situation. However, care is not uniform across medical centers, even in the ICU dataset. Additionally, the future trajectory might depend on risk factors such as age, sex or existing conditions. Ideally, future works should try to add current treatment information and clinically relevant patient information to the prognosis model, a step towards personalized medicine. Another limitation is the exclusion of ‘Stable’ image pairs in the prediction of the radiological trajectory. Their exclusion was initially justified by their small number in the COVID-19 image data collection, however as seen in the ICU dataset they actually represent a large proportion of the typical radiological trajectory. Next, the medical assessment of severity and progression was performed before the staging system introduced by Wong et al.^8^ and used by Cohen et al.^23^. This limits the inter-work comparison, even if it does not impact the conclusions put forward in this work. The closest work, only in terms of severity evaluation, is the work of Blain et al.^25^, who report severity on a 0–3 scale similar to the one used in this work. They report a 78% accuracy in the 4-way classification of severity, higher than ours of 52.3%. However, due to their small dataset size (65 CXRs), this performance was only cross-validated, and not tested. Moreover, a end-to-end deep learning model was used for this task, leading to concerns of overfitting. Finally, the design of the study is retrospective. However, as the pandemic unfolds, new clinical and radiological data will be continuously incorporated in the test set from the open source repositories, and from additional cases from the authors’ institutions, which will truly test generalizability and solve some of these limitations.

To a degree, this report has confirmed that deep learning features can track radiological progression in COVID-19 but also predict temporal evolution, adding evidence to the conceptualization that there is directional information in static X-rays allowing this prediction. However, it should be restated that the reference standard was categorization of imaging rather than clinical outcomes, such as duration of ICU stay or mortality. Further studies should therefore assess the added value of deep learning features in clinical decision making using multivariate models incorporating additional variables such as vital signs, oxygenation and ventilation parameters, and assessment of imaging data, such as the reported hazard ratios published in large scale studies^34^.

## Conclusion

We found that deep learning was able to assess the severity and predict the radiological evolution of the SARS-CoV-2 over CXR images. Patients at risk of a worsening radiological presentation over a short period could be identified in both a ICU-bound, intubated population and in a more general hospital-admitted population. Automated analysis of CXRs for COVID-19 afflicted patients has the potential to alleviate the burden of medical providers and inform future treatment by identifying at-risk patients and by staging the current severity of the disease. Even though concerns of bias, overfitting and distribution shift remains, deep learning could be a critical tool to help in patient management and also in ressource-allocation scenarios such as triage. Shared decision making integrating the best available prognostic models, clinician experience and patient values and preferences about life-sustaining therapies will also be paramount in making these very difficult decisions.

## Supplementary Information

Supplementary Information.
